# Testicular Mass: An Initial Sign of Squamous Cell Carcinoma of the Lung

**DOI:** 10.4021/wjon568w

**Published:** 2013-01-04

**Authors:** Muhammet Ali Kaplan, Mehmet Kucukoner, Ali Inal, Zuhat Urakci, Ugur Firat, Halil Komek, Abdurrahman Isikdogan

**Affiliations:** a Department of Medical Oncology, Dicle University, 21280, Diyarbakir, Turkey; b Department of Pathology, Dicle University, 21280, Diyarbakir, Turkey; c Department of Nuclear Medicine, Diyarbakir State Hospital, 21280, Diyarbakir, Turkey

**Keywords:** Testicular metastasis, Lung cancer, Squamous cell

## Abstract

Metastatic carcinoma to the testis, excluding lymphoma and leukemia, is an extremely rare condition. The most frequent primary site is prostate cancer. These lesions present clinically either as the first sign of malignancy or more commonly as a complication during the course of known disease. We present the first case of a squamous cell carcinoma of the lung diagnosed with a testicular mass, which is very rarely seen in literature, to our knowledge.

## Introduction

Although testicular neoplasms account for 1% of all cancers, germ cell testical tumors are the most common solid tumors among men between the ages of 20 and 40 years [1]. Majority of testicular neoplasms are primary tumors, but metastasis to testicles is very rare, excluding lymphoma and leukemias. More than 200 reported cases were detected incidentally during autopsies or during orchiectomy for the treatment of prostate cancer [2]. However, approximately 6-7% of testicular neoplasms present as mass, and there is no lung cancer cases diagnosed with testicular mass although cases with testicular metastasis from lung cancer are available in the literature [2-4]. The adenocarcinoma of the prostate gland is the most common primary tumor whereas only a very small number of squamous cell carcinoma of the lung has been reported [5, 6]. We present the case of a squamous cell carcinoma of the lung diagnosed with a testicular mass, which is very rarely seen in literature.

## Case Report

**Figure 1:**
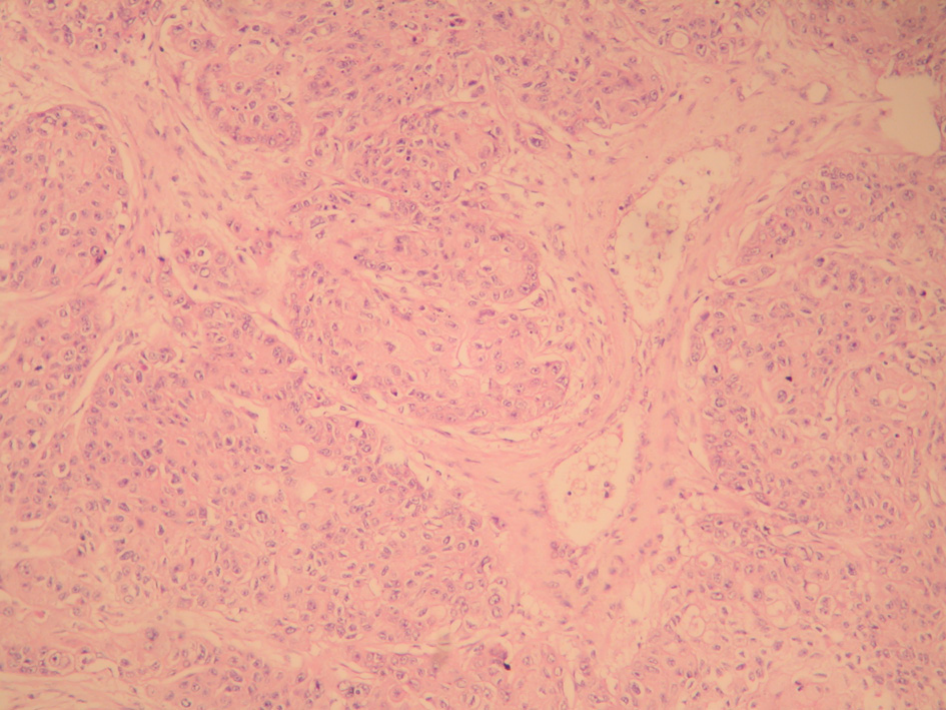
Malignant epithelial tumor infiltration within the interstisium of the testicular parenchima (H&E stain, × 100).

**Figure 2:**
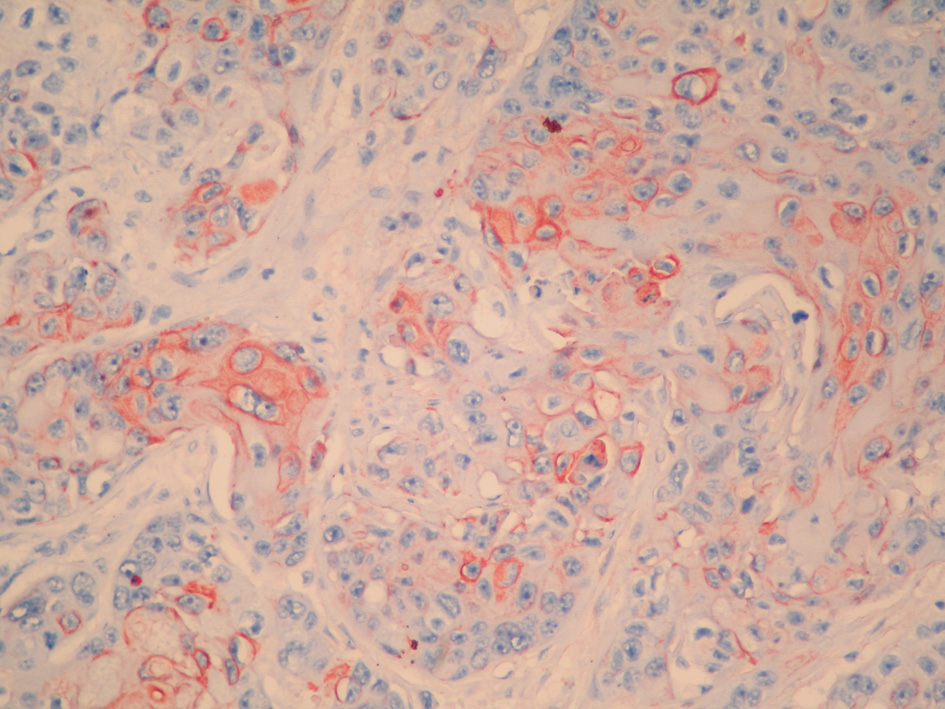
Cytokeratin 19 positivity in the tumor cells (Immunoperoxidase, × 400).

**Figure 3:**
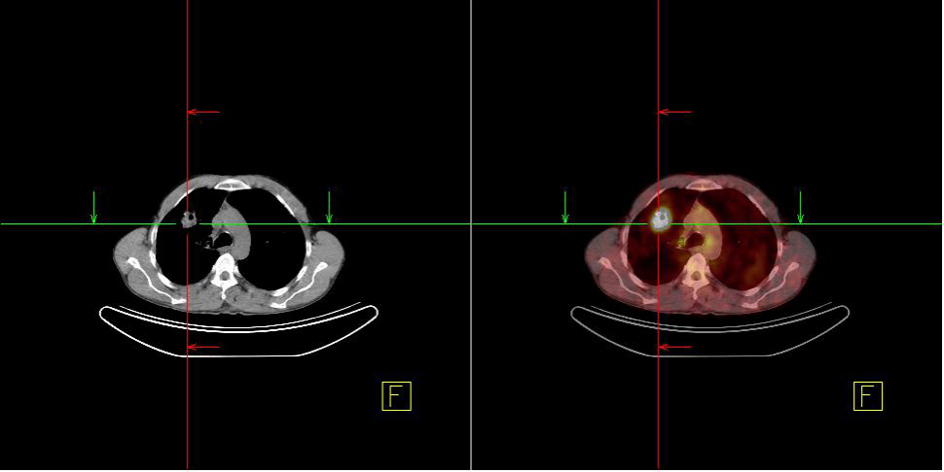
Lung mass detected in the PET/CT imaging.

A 48-year-old male patient presented to the urology clinic for painless swelling of his right testis that has been going on approximately for two months. The patient had 60 pack/years of smoking history, and considering presence of a primary testicular tumor, he was scheduled for a surgery by the urology department. His pre-operative B-HCG and AFP levels were assessed, and found within acceptable limits, and he underwent orchiectomy. The immunohistochemical staining performed for the patient, who had a manifestation consistent with squamous cell cancer during the pathological assessment showed focal CK19 and keratin positivity as well as PLAP, AFP, CD30, CK5/6, HMWCK, TTF-1, HMB45 AND Melanin A negativity (Fig. 1 and 2). After the histological subtype was identified as squamous cell carcinoma, PET/CT imaging that was performed to detect the primary disease revealed a 3.5 cm malignant tumor in the right lung with an SUVmax of 9, metastatic nodules in bilateral lungs with an SUVmax of 2.6, and bone metastases in the right supraclavicular, upper right paratracheal, aorticopulmonary, subcarineal and left hiler lymph nodes with an SUVmax of 13, in the right surrenal gland with an SUVmax of 9.4, and in the lumbar vertebrae and left iliac bone with an SUVmax of 4 (Fig. 3). Considering squamous cell carcinoma of the lung as a primary focus, a palliative treatment was initiated with Gemzar (1,000 mg/m^2^, on day 1 and day 8, every 3 weeks), Cisplatin (75 mg/m^2^, on day 1, every 3 weeks), and zoledronic acid (4 mg/day, every 3 weeks).

## Discussion

Testicular metastasis is a very rare condition. While incidence of metastatic testicular cancer ranges from 0.06% to 0.46% in large unselected autopsy series, its ratio among all testical tumors varies between 0.8% and 2.3% [2, 7]. In a literature review by Patel et al in 1989, majority of 209 cases were either from autopsy series and from prostate cancer cases who underwent orchiectomy for hormonal therapy [2]. Of these 209 cases, only 13 (6.2%) had presented with testicular tumor, and had no lung cancer [2]. They reported that the most common primary was tumors of prostate (34.6%), followed by neoplasms of lungs (17.3%) [2]. During our literature review, we found that there are 9 cases of lung cancer reported after this publication in 1989. Three of these cases were from autopsy series [7] and 5 of the remaining cases occurred approximately from 2 weeks to 2 years after diagnosis [5, 6, 8-10] while only one adenocarcinoma case presented with testicular tumor at the time of diagnosis [6]. To our knowledge, among 40 lung cancer cases reported, only one had adenocarcinoma presented with a testicular tumor at the time of diagnosis whereas our case is the first squamous cell cancer patient who presented with a testicular tumor at the time of diagnosis. Furthermore another distinguishing feature of our case is that our patient’s complaint was only of testicular tumor at the time of diagnosis and the diagnosis was made based on orchiectomy. In conclusion, it should be kept in mind that patients who present with testicular tumors may have a metastasis secondary to an underlying malignity although it is very rare.
